# Effects of Water Deficit and GABA-Containing Biostimulant on Maize Plants: Nondestructive Monitoring by X‑Ray Fluorescence and Visible Spectroscopy

**DOI:** 10.1021/acsomega.6c00213

**Published:** 2026-06-02

**Authors:** João Pedro Chacon Pereira, Fábio Luiz Melquiades, José Vinícius Ribeiro, Gabriela Machineski, Inês Cristina de Batista Fonseca, Halley Caixeta Oliveira

**Affiliations:** † Department of Agronomy, Postgraduate Program in Agronomy, State University of Londrina (UEL), Londrina 86057-970, Paraná, Brazil; ‡ Department of Physics, State University of Londrina (UEL), Londrina 86057-970, Paraná, Brazil; § Department of Animal and Plant Biology, State University of Londrina (UEL), Londrina 86057-970, Paraná, Brazil

## Abstract

Climate change increases the frequency of water deficit in crops, requiring efficient mitigation and monitoring strategies. This study evaluated the use of portable X-ray fluorescence (pXRF) and visible spectroscopy (400–700 nm), combined with principal component analysis (PCA), to rapidly detect water stress in maize (*Zea mays* L.), and assessed the protective effect of a foliar biostimulant containing GABA (GCB). Water deficit reduced stomatal conductance, chlorophyll content, and plant growth, while increasing membrane damage and leaf temperature. GCB, especially at 30 g ha^–1^, mitigated stress effects by improving physiological stability, preserving photosynthetic pigments, and enhancing biomass and leaf area. PCA effectively discriminated treatments, with 670–690 nm (chlorophyll) and 490–600 nm (carotenoids) identified as key spectral regions, while pXRF signals for K and Ca reflected stress-induced changes. The integration of biostimulants with portable spectroscopy represents a promising nondestructive approach for monitoring and managing water stress in maize under climate-related constraints.

## Graphical abstract

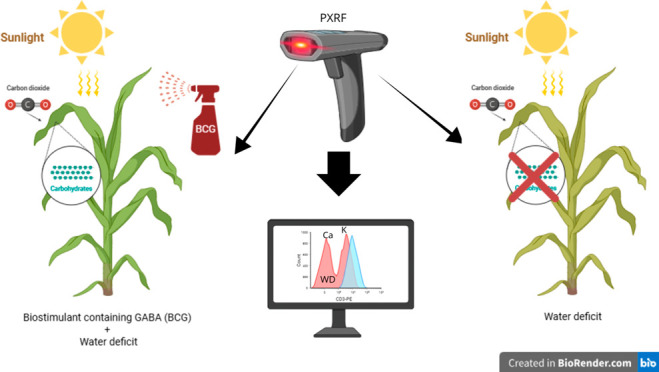

## 1 Introduction

Climate change is one of the most significant threats to global food security, driving shifts in precipitation patterns and increasing the frequency and intensity of extreme events such as droughts and heatwaves, factors that severely impact agricultural systems worldwide.^1^ Under drought conditions, maize (*Zea mays* L.), a crop of major economic and strategic importance for food and bioenergy production, often exhibits substantial declines in vegetative growth, photosynthetic efficiency, and yield.^2,3^

Considering these challenges, the development and implementation of efficient plant water status monitoring strategies are essential to minimize yield losses. Nondestructive and early detection techniques, such as spectral reflectance,^4^ optical modeling using stress-sensitive spectral indices,^5^ stem electrical impedance analysis,^6^ and terahertz radiation imaging^7^ have demonstrated strong potential for detecting water stress in plants. Early diagnosis of stress allows for timely deployment of mitigation tools, such as biostimulants, which are promising yet complex agronomic inputs that are capable of modulating key physiological and biochemical defense mechanisms in plants.^8^

Among these biostimulants, gamma-aminobutyric acid (GABA) has gained attention due to its signaling function in plants, particularly under abiotic stress. GABA plays a crucial role in osmotic adjustment, regulation of stomatal conductance, and activation of antioxidant defense systems.^9,10^ Exogenous GABA applications have been shown to induce stomatal closure, reduce transpirational water loss, increase relative water content, and preserve membrane integrity, thereby alleviating the physiological damage caused by drought.^11,12^ Moreover, GABA enhances the synthesis of osmolytes, such as proline, and soluble sugars, supporting photosynthesis and cellular turgor maintenance. It also stimulates the activity of antioxidant enzymes, including superoxide dismutase (SOD), ascorbate peroxidase (APX), and catalase (CAT), reducing reactive oxygen species (ROS) accumulation and lipid peroxidation.^13,14^

Recent research has further explored the foliar application of other biostimulants, such as protein hydrolysates (PHs) and seaweed extracts, that are capable of enhancing crop resilience under abiotic stress.^14,15^ These products supply amino acids and bioactive peptides that regulate nitrogen metabolism and activate signaling pathways related to growth and stress tolerance, often displaying hormone-like activity and synergistic effects when combined with other biostimulants.^16,17^ In particular, seaweed extracts are a natural source of phytohormones (auxins, cytokinins, gibberellins, and triacontanol), betaines, and phenolic compounds, that contribute to root growth stimulation, photosynthetic protection, and antioxidant regulation under stress.^18,19^ These findings highlight the importance of considering not only GABA but also the combined and potentially synergistic effects of other bioactive components present in commercial formulations.^20^ Simultaneously, other innovative approaches, such as machine learning-assisted data analysis and nondestructive sensing technologies, have emerged as promising strategies to improve the early detection and management of environmental stress in a more targeted and sustainable manner.^21^

Within the framework of precision agriculture, rapid, sensitive, and noninvasive monitoring tools are indispensable for real-time assessment of plant physiological responses. Optical techniques, including visible spectroscopy (400–700 nm), can detect spectral changes linked to plant stress responses. Portable X-ray fluorescence spectrometry (pXRF) also offers valuable insights, by detecting changes in leaf mineral composition before visual symptoms become apparent.^13,22^ When combined with multivariate statistical tools such as Principal Component Analysis (PCA), spectral data sets can be leveraged to differentiate plant water status even across genetically distinct cultivars.^24,25^

In this context, we aimed to (i) evaluate the physiological effectiveness of a GABA-containing biostimulant (GCB), a composite formulation including GABA, seaweed-derived growth regulators, and protein hydrolysates, in mitigating drought stress in maize, and (ii) determine whether portable X-ray fluorescence (pXRF) and spectrometry visible spectroscopy (400–700 nm), combined with multivariate analyses, can detect drought and GCB treatment effects through a rapid, early, and nondestructive monitoring approach. Our central hypotheses were: (i) GCB mitigates drought effects in maize through multicomponent action; (ii) spectral monitoring platforms enable early and precise detection of stress responses, supporting more resilient and sustainable agricultural practices.

## 2 Materials and Methods

### 2.1 Experimental Site and Greenhouse Conditions

The experiment was conducted in a greenhouse at the Agrarian Sciences Center of the State University of Londrina (UEL), Londrina, Paraná, Brazil (23°20′23.45″ S; 51°12′32.28″ W; 560 m altitude). The structure enabled partial control of temperature and humidity and was equipped with an automated irrigation system, using sprinkler nozzles to simulate rainfall, ensuring uniform water distribution. Water management consisted of scheduled daily irrigation for 25 min in the morning (07:00–07:25) and 10 min in the afternoon (12:15–12:25). The maize hybrid (*Z. mays* L.) FS552 PWU was grown in 20 L pots filled with a Rhodic Kandiudalf soil (Eutroferric Latosolic Red Nitosol), previously characterized and corrected according to crop requirements.^26^ The soil chemical analysis showed 10.3 g dm^–3^ of organic matter, a pH (CaCl_2_) of 4.8, 11.6 mg dm^–3^ of P, 0.53 cmol_c_ dm^–3^ of K^+^, 6.33 cmol_c_ dm^–3^ of Ca^2+^, 1.5 cmol_c_ dm^–3^ of Mg^2+^, and base saturation of 67.9%. Fertilization was defined based on the soil analysis results and maize crop recommendations, considering the estimated extraction of 80.5 kg ha^–1^ of N, 8.7 kg ha^–1^ of P, and 31.8 kg ha^–1^ of K per ton of grain.^27^

### 2.2 Experimental Design

The experiment was conducted in a completely randomized design (CRD) in a 2 × 3 factorial scheme, with nine replications, totaling 54 experimental units. The first factor consisted of two water regimes: well-watered plants, maintained under optimal soil moisture through an automated sprinkler system with rain-simulator nozzles, and drought-stressed plants, subjected to irrigation suspension for 15 consecutive days. Stress progression was monitored visually during the experimental period and confirmed by physiological parameters. The second factor comprised the foliar application of a GABA-containing biostimulant (GCB) at three concentrations: 0 g ha^–1^ (control-water only), 30 g ha^–1^ (moderate dose), and 60 g ha^–1^ (high dose). Both the GCB application and the initiation of the drought treatment were performed at the V4 stage, identified according to the criteria for fully expanded leaves described by Ciampitti et al.^28^ Foliar applications were carried out using a CO_2_-pressurized backpack sprayer calibrated for a spray volume of 100 mL ha^–1^.

The GCB formulation contained seaweed extract as a source of plant growth regulators (6-benzylaminopurine −100 mg L^–1^, 2-naphthoxyacetic acid −50 mg L^–1^, naphthaleneacetic acid −50 mg L^–1^, indole-3-acetic acid −50 mg L^–1^, and triacontanol −20 mg L^–1^), supplemented with hydrolyzed bovine collagen obtained from the chemical hydrolysis of leather industry byproducts. The product was manufactured in Brazil by Nutriten (Brazil). The amino acid profile (e.g., glycine 13.5%, glutamic acid 5.6%, proline 8.3%) is presented in Supporting Information Table S1.

### 2.3 Portable X-Ray Fluorescence Analysis

At the end of the 15 day water deficit period (or on the same day for well-watered plants), pXRF measurements were performed directly on the leaves, without prior preparation, and with standardized reading time per sample, to extract the elemental spectral patterns. A hand-held Tracer 5i (Bruker Inc.) pXRF instrument was used, inside the vegetation house in the benchtop configuration. Each sample was positioned near the instrument for measurement. The experimental conditions were set to 30 kV, 13 μA, and 30 s, using 25 μm Ti and 300 μm Al filters to excite a range of plant-relevant elements, as well as Compton and Rayleigh scattering signals. Measurements were performed directly on intact maize leaves using a portable support stand to maintain constant positioning between the sensor and the sample. The leaf blade was placed over the instrument reading window, and an acrylic plate with a central opening was positioned over the leaf surface to stabilize the sample during acquisition. The shield cap was used for radioprotection purposes. Figure S1 presents photographs of the measurement setup and procedures.

### 2.4 Colorimetric Analysis

The colorimetric evaluation was conducted using the NIX Pro 2 digital colorimeter as a complementary tool for comparison with pXRF. Analogously to the pXRF measurements, the instrument was placed directly on the leaves, with an acrylic support in the back for mechanical stability of the instrument in hand, and all readings were performed at the end of the 15 day water deficit period (or on the same day for well-watered plants). The illuminant D50 2° was configured on the equipment, to record spectra data from 400 to 700 nm and compute the Lab (L, a, b), LCh (c, h), XYZ (X, Y, Z), and sRGB (sRGB R, sRGB G, sRGB B) color scales. The measurement time was less than 2 s and three replicates per leaf were extracted.

### 2.5 Physiological and Biometric Parameters

Physiological evaluations were conducted after 15 days of water deficit, using the second fully expanded youngest leaf of each plant. Chlorophyll content was measured with a chlorophyll meter (Falker model CFL1030, Brazil), including chlorophyll *a* (Chl *a*), chlorophyll *b* (Chl *b*), total chlorophyll (Chl t = Chl *a* + Chl *b*), and the chlorophyll *a*/*b* ratio (RChl *ab* = Chl *a*/Chl *b*).

Leaf temperature (LT, °C) was determined using an industrial infrared thermal camera (UTi-260B Uni-Tpro 550 °C, Uni-Trend, China), with three readings per leaf, averaged to obtain a single value per plant. Stomatal conductance (*g*_s_ mol m^–2^ s^–1^) was measured on the abaxial surface of the leaf using a SC1 porometer (Decagon Devices, Pullman, WA, USA).

Cell membrane integrity was evaluated by relative electrolyte leakage (EL, %). Leaf discs (0.75 cm diameter, ∼0.2 g) were incubated in demineralized water for 6 h at room temperature to determine initial electrical conductivity (*CE*_i_), then heated at 90 °C for 2 h and cooled to obtain final conductivity (*CE*_*f*_). Electrolyte leakage was calculated using the following eq 1

1 $$EL\;\left(\%\right)=\frac{{CE}_{i}}{{CE}_{f}}\times 100$$

Relative water content (RWC, %) was determined according to the method of Smart and Bingham.^29^ Leaf samples of approximately 50 mg were collected, submerged in distilled water for 24 h, and weighed to obtain turgid mass (TM). Samples were then oven-dried at 60 °C for 72 h to determine dry mass (DM). Leaf RWC was calculated using the following eq 2

2 $$RWC\;\left(\%\right)=\frac{Fresh\;mass-Dry\;mass}{Turgid\;mass-Dry\;mass}\times 100$$

Vegetative growth variables were assessed at the same time as the physiological measurements, when the plants were cut at soil level and removed from the pots. Shoot length (SL, cm) and root length (RL, cm) were measured using a millimeter tape, and stem diameter (SD, mm) was measured with a 150 mm stainless-steel digital caliper (MTX, Brazil). Shoot fresh mass (SFM, g) and root fresh mass (RFM, g) were obtained by separating the tissues and weighing them on a semianalytical scale (SAUTER RC 2013, Germany). Leaf area (LA, cm^2^) was determined using an LI-3000C electronic area meter (LI-COR, Lincoln, NE, USA). Subsequently, samples were dried in a forced-air oven at 60 °C for 72 h to determine shoot dry mass (SDM, g) and root dry mass (RDM, g). Water loss percentage was calculated as the difference between fresh and dry mass. All measurements were performed on nine plants per treatment.

### 2.6 Statistical Analysis

The data obtained in the experiment were initially analyzed to test the basic assumptions necessary for applying parametric methods through the Shapiro–Wilk normality test and the Hartley homogeneity test. Once adherence was confirmed, analysis of variance (ANOVA) was performed using the F-test, with a 5% significance level (*p* < 0.05), to evaluate the main effects of water deficit and GCB application, as well as their interaction, on the morphological and physiological variables of corn. When significant differences were identified, the means of the treatments were compared using the Tukey test. Additionally, regression models were adjusted for the quantitative variables to describe response trends to the treatments. All analyses were performed via R software (version 4.4.3) in conjunction with the AgroR package,^30^ which specializes in statistical procedures applied to agricultural experimentation.

For the multivariate analysis, the spectral data obtained both by pXRF and by the NIX colorimeter were preprocessed appropriately considering the nature of each set. While the pXRF data underwent mean centering, the visible reflectance spectra were smoothed by applying the Savitzky–Golay (window = 13, polynomial degree = 2, and derivative order = 1) and multiplicative scatter correction methods and then mean centered. The colorimetric parameters were scaled by standard deviation (autoscaling). Subsequently, Principal Component Analysis (PCA) was applied to reduce data dimensionality, identify clustering patterns among treatments, and determine the variables with higher contribution to sample discrimination. Principal components were selected based on their cumulative explained variance and graphical interpretability. Treatment groupings were interpreted according to the spatial proximity of samples in the score plots, where treatments with similar multivariate responses were positioned closer together, while more distinct treatments were separated along the principal component axes. PCA analyses were performed in the R environment using the mixOmics, AgroR, and ggplot2 packages for data processing and generation of score and loading plots.

## 3 Results and Discussion

### 3.1 Morphophysiological and Biometric Parameters

Plants under water deficit (WD) showed symptoms of reduced growth and leaf expansion compared to those under field capacity (Figure 1). Indeed, the ANOVA revealed that WD significantly reduced SD, LA, RDM, and SDM, highlighting the structural damage caused by limited water availability to maize development (Table 1). These reductions are consistent with the physiological constraints imposed by drought, which negatively affect nutrient uptake and photosynthesis, and assimilate transport, ultimately leading to lower biomass allocation.^31,32^ Spectral evidence, such as that reported by Baranoski,^5^ also indicates that these changes are preceded by measurable optical signals, underscoring the importance of early detection to mitigate functional and yield losses.

**Figure 1:**
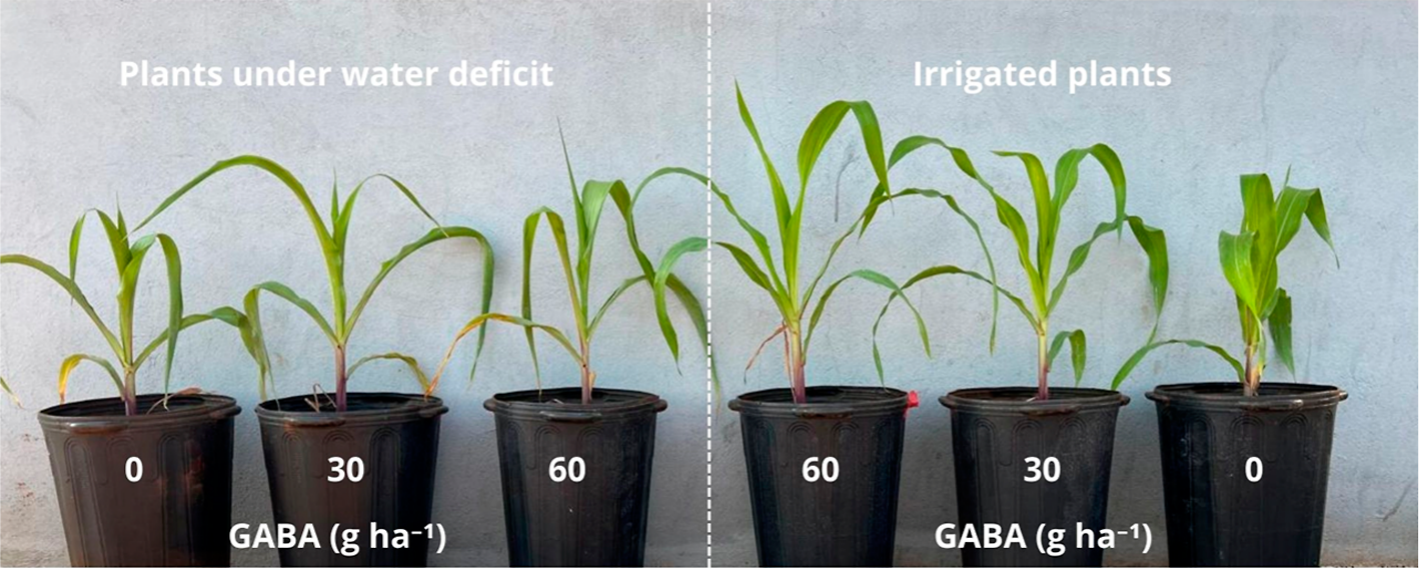
Visual appearance of maize plants subjected to full irrigation or water deficit under foliar application of γ-aminobutyric acid (GCB) biostimulant at 0, 30, and 60 g ha^–1^.

**Table 1:** Analysis of Variance (ANOVA) of the Effects of Water Deficit (WD) and Foliar Application of a Biostimulant Containing γ-aminobutyric Acid (GCB; 0, 30 and 60 g ha^–1^) on Stem Diameter (SD), Leaf Area (LA), Root dry Mass (RDM), Shoot dry Mass (SFM) Root Length (RL) and Shoot Length (SL) of Maize Plants^a^ ^b^ ^c^

causes of variation	D.F	*p*-Value					
		SD (mm)	LA (cm^2^)	RDM (g)	SDM (g)	RL (cm)	SL (cm)
Water deficit (WD)	1	0.0515*	0.0001*	0.3062	0.0001*	0.4534	0.4574
GCB (G)	2	0.0001*	0.0001*	0.0528	0.0009*	0.9554	0.4409
DH x G	2	0.0776	0.4498	0.4366	0.0835	0.5991	0.7344
Residuals	42	-	-	-	-	-	-
C.V. (5%)		19.51	16.04	17.43	20.21	21.17	18.12
Overall mean		1.23	465.54	2.07	9.09	27.02	28.74
Irrigated		1.26 a	588.11 a	2.17 a	11.93 a	25.50 a	65.18 a
under WD		1.22 b	238.83 b	1.97 b	6.26 b	27.38 b	58.32 b

a Significant effects are indicated by * at *p* ≤ 0.05.

b Means followed by different letters within each column differ significantly according to Tukey’s test (*p* ≤ 0.05).

c Values represent mean ± standard deviation.

Foliar application of GCB was associated with improved plant growth traits, while water deficit reduced overall plant vigor. Although the WD × GCB interaction was not significant, plants treated with GCB showed a better growth response across water conditions (Figure 1 and Table 1).

The application of GCB significantly increased SDM and SD irrespective of water availability (Figure 2). In the control, SDM averaged 5.78 g, whereas applications of GCB at 30 and 60 g ha^–1^ raised this value to 11.49 and 10.01 g, respectively, indicating a plateau consistent with a physiological saturation point. While these biomass gains align with known roles of GABA in modulating energy metabolism, osmolyte synthesis, and anabolic pathways under stress,^10,33^ the tested product is a composite containing seaweed-derived growth regulators and protein hydrolysate; thus, the responses likely reflect the combined and potentially synergistic actions of these constituents.^9,16–18^

**Figure 2:**
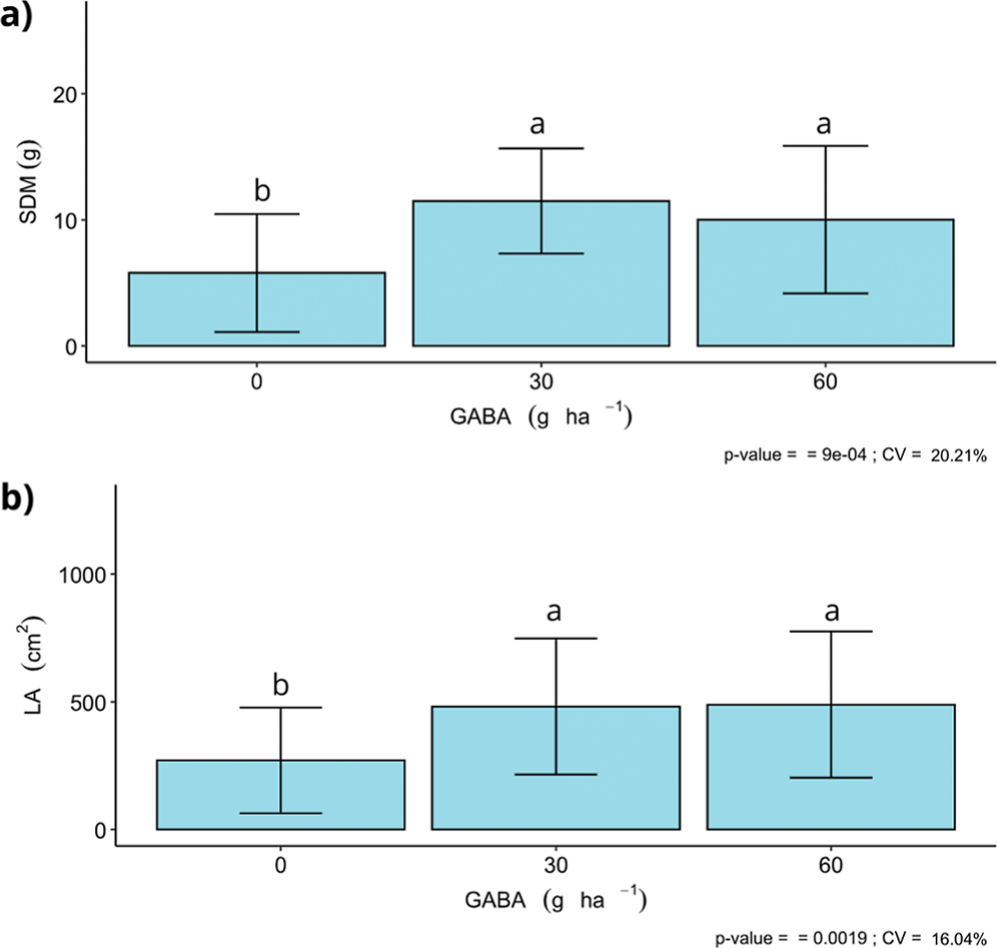
Effects of foliar application of a biostimulant with different concentrations of a biostimulant containing γ-aminobutyric acid (GCB; 0, 30 and 60 g ha^–1^) on shoot dry mass (SDM) (a) and leaf area (LA) (b) of maize plants. Data are presented as mean ± standard deviation.

The increase in stem diameter (from 1.01 mm in the control to 1.34 and 1.36 mm with 30 and 60 g ha^–1^, respectively) may therefore reflect not only GABA-mediated protection but also structural reinforcement, promoted by hormone-like signals from seaweed extracts and bioactive peptides/amino acids from protein hydrolysates, which can stimulate secondary cell wall deposition and lignification mechanisms aligned with improved mechanical resistance and canopy stability.^9,11,12,18^

Additionally, the increase in the leaf area index (LAI), from an average of 271 (control) to 481 (30 g ha^–1^) and 489 (60 g ha^–1^), highlights the role of GCB in preserving cellular turgor, enabling leaf elongation, and maintaining plant architecture under water deficit. This pattern is consistent with the stomatal and osmotic regulation described for GABA.^13,15^ and with the expected hormone-like and nutritional contributions of seaweed extracts and protein hydrolysates under drought, as shown in maize at the transcriptomic/metabolomic level^19,20^ and in crops treated with PH-seaweed blends.^18^

It is also worth noting that similar results in promoting growth and leaf expansion were reported by Barbosa da Silva et al.^14^ in studies involving other biostimulants, such as seaweed extracts and plant growth regulators. These findings reinforce the effectiveness of biostimulant-based approaches. Thus, GCB should not be seen solely as a stress mitigator, but also as a growth promoter under diverse environmental conditions, positioning it as a versatile tool for optimizing light capture, photosynthetic efficiency, and productivity in water-limited agricultural systems.

These results reinforce the potential of GCB as a multifunctional formulation capable of contributing to cellular turgor maintenance, osmotic balance, and stress mitigation under water deficit conditions.^11,34^ Similar responses have been reported for other biostimulant formulations, which promoted reductions in transpirational water loss and greater biomass accumulation under adverse conditions.^15^ Thus, the effects observed in the present study indicate that GCB may act through comparable physiological mechanisms, contributing to improved plant performance under drought.

Structurally, GCB exhibited the ability to stimulate lignin biosynthesis, thereby enhancing stalk reinforcement and the mechanical support of the leaf canopy.^12^ Given the composite nature of the product, this reinforcement is also likely driven by hormone-like compounds from seaweed extracts and bioactive peptides from protein hydrolysates, which promote secondary cell wall deposition and stress-induced fortification.^9,16,17^ This is particularly beneficial in maize, where stalk stability directly affects light interception and lodging resistance.

The ANOVA, complemented by mean comparison analysis, confirmed that WD had a highly significant effect on all evaluated physiological parameters (Table 2). Leaf RWC, *g*_s_ and chlorophyll level were reduced, whereas LT and EL increased, characterizing a typical physiological response to severe water deficit and validating the stress severity imposed in the experiment.

**Table 2:** Analysis of Variance (ANOVA) of the Effects of Water Deficit (WD) and Foliar Application of a Biostimulant Containing γ-Aminobutyric Acid (GCB; 0, 30 and 60 g ha^–1^) on Physiological Parameters of Maize Plants: Adventitious Root Length (ARL), Photosynthetic Rate (A), Membrane Integrity (MI), Chlorophyll *b* Content (Chl *b*), Chlorophyll a Content (Chl *a*), Total Chlorophyll (Chl t), Chlorophyll *a/b* Ratio (Chl *a/b*) and Stomatal Conductance (*g*_s_)­^a^ ^b^ ^c^

Causes of variation	D.F	*p*-Value							
		RWC (%)	LT (°C)	EL (%)	Chl *b*	Chl *a*	Chl t	RChl *ab*	*g*_s_ (mmol m^2^ s^–1^)
Water deficit (WD)	1	0.0289*	0.0001*	0.0001*	0.0012*	0.0004*	0.0005*	0.0293*	0.0004*
GCB (G)	2	0.586	0.0893	0.0001*	0.0465*	0.0209*	0.0251*	0.1545	0.8634
WD x G	2	0.725	0.1423	0.0922	0.9827	0.9773	0.9786	0.8109	0.009*
Residuals	42	-	-	-	-	-	-	-	-
C.V. (5%)		20.14	9.62	62.78	20.69	13.78	15.16	7.82	28.52
Overall mean		63.21	32.32	24.58	7.35	25.78	33.14	3.56	226.27
Irrigated		65.18 a	28.77 b	12.50 b	27.75 a	8.11 a	35.86 a	3.65 a	262.03 a
under WD		58.32 b	33.89 a	36.67 a	23.83 b	6.60 b	30.43 b	3.47b	190.52 b

a Significant effects are indicated by * at *p* ≤ 0.05.

b Means followed by different letters within each column differ significantly according to Tukey’s test (*p* ≤ 0.05).

c Values represent mean ± standard deviation.

Graphical analysis showed that GCB application significantly influenced Chl t, Chl *a*, and Chl *b* levels (Figure 3), with progressive increases observed at the 30 and 60 g ha^–1^ doses. Chl t values rose from 30.3 in the control to 33.9 and 35.2, respectively. Similar increments were observed for Chl *a* (23.7; 26.4; 27.3) and Chl *b* (6.59; 7.53; 7.94), all statistically significant. These results indicate that the biostimulant exerted a direct protective effect on photosynthetic pigments, attenuating their degradation even under drought stress, an outcome likely reflecting the combined action of GABA, amino acids, and seaweed-derived phytohormones present in the formulation.^16,19,20^

**Figure 3:**
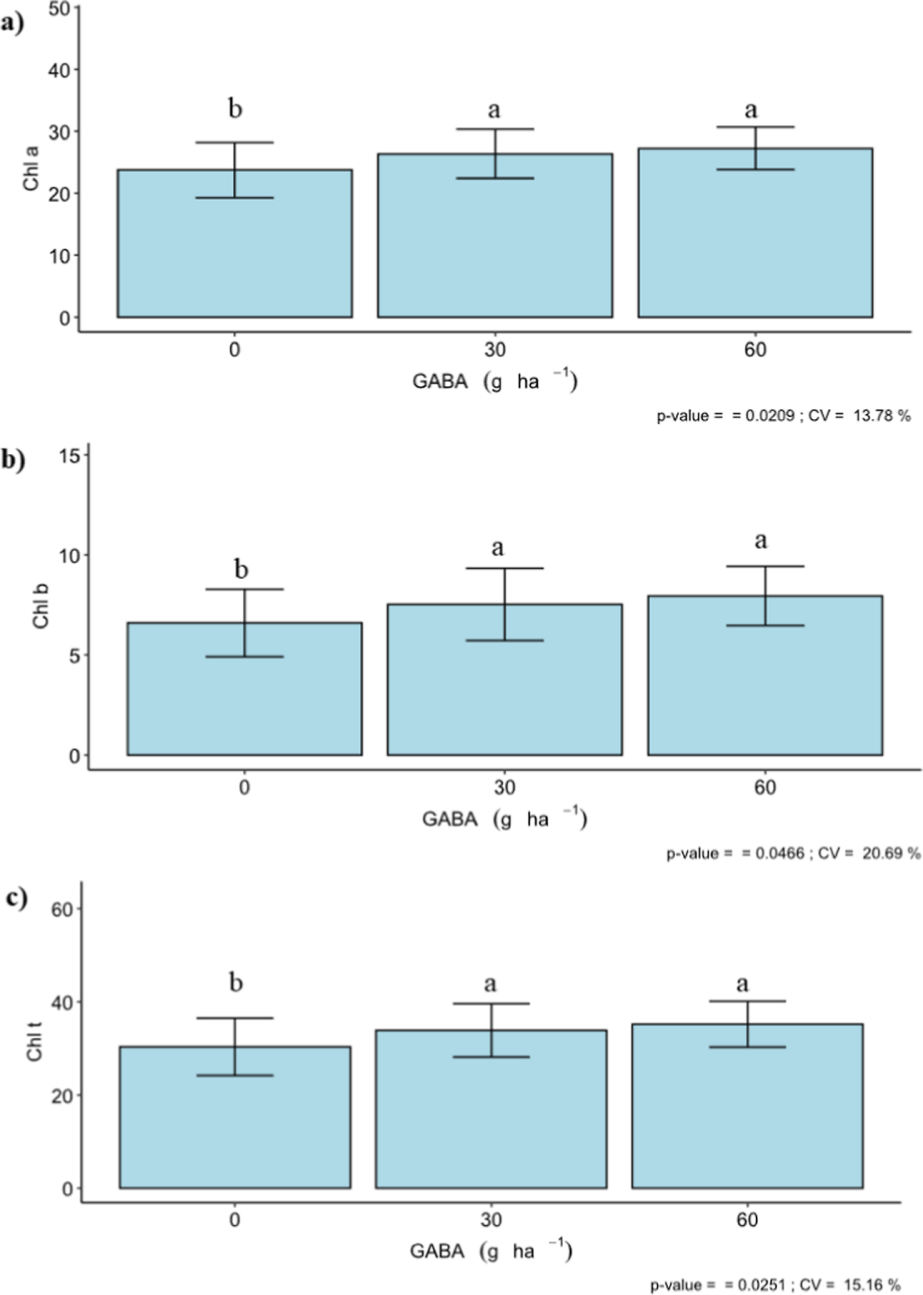
Effects of foliar application of a biostimulant containing γ-aminobutyric acid (GCB; 0, 30 and 60 g ha^–1^) on chlorophyll *a* (Chl *a*) (a), chlorophyll *b* (Chl *b*) (b) and total chlorophyll (Chl t) (c) contents of maize plants under water deficit. Data are presented as mean ± standard deviation.

This protection is attributed to the ability of GABA to mitigate ROS by enhancing enzymatic antioxidant systems (SOD, CAT, POD), thereby preserving thylakoid integrity and maintaining PSII functionality.^10,15^ In a composite formulation, seaweed-derived phytohormones and amino-acid peptides can further up-regulate genes and pathways involved in chlorophyll biosynthesis, ROS scavenging, and photoprotection.^16,19,20^ Pigment integrity is essential not only for photosynthesis but also for maintaining leaf reflectance in the red and near-infrared spectrum, which directly affects early stress detection through optical monitoring, as demonstrated in spectral simulation models by Baranoski^5^ and in visible range (400–700 nm) analyses by Patiluna et al.^24^

Parallel studies support these findings. Geng et al.^11^ demonstrated that GABA preserved chlorophyll levels in sunflowers under combined drought and heat stress, resulting in improved light use efficiency and photosynthetic rate. Similarly, Seifikalhor et al.^10^ reported enhanced pigment stability and antioxidant activity in GCB-treated chickpeas, with a significant reduction in lipid peroxidation.

The stronger response observed at the 60 g ha^–1^ dose suggests a physiological threshold, wherein GCB operates both to mitigate oxidative damage and to stabilize the structure of light-harvesting complexes (LHC), ensuring greater photosynthetic efficiency under drought conditions. This dual role, protective and promotive, positions GCB as a strategic tool to sustain photosynthetic performance and crop productivity in stress-prone environments.

The increase in *g*_s_ following the application of 30 and 60 g ha^–1^ of GCB, particularly under water deficit (Figure 4), indicates a positive effect on stomatal regulation. This response has been associated with ABA signaling modulation.^10,15^ In the present study, treated plants exhibited mean *g*_s_ values of 209 and 217 μmol m^–2^ s^–1^, significantly higher than the control (146 μmol m^–2^ s^–1^), suggesting stress-dependent benefits. Hormonal crosstalk and osmotic adjustment arising from the seaweed and protein hydrolysate components likely contributed to the observed gains in *gs*.^17,18^

**Figure 4:**
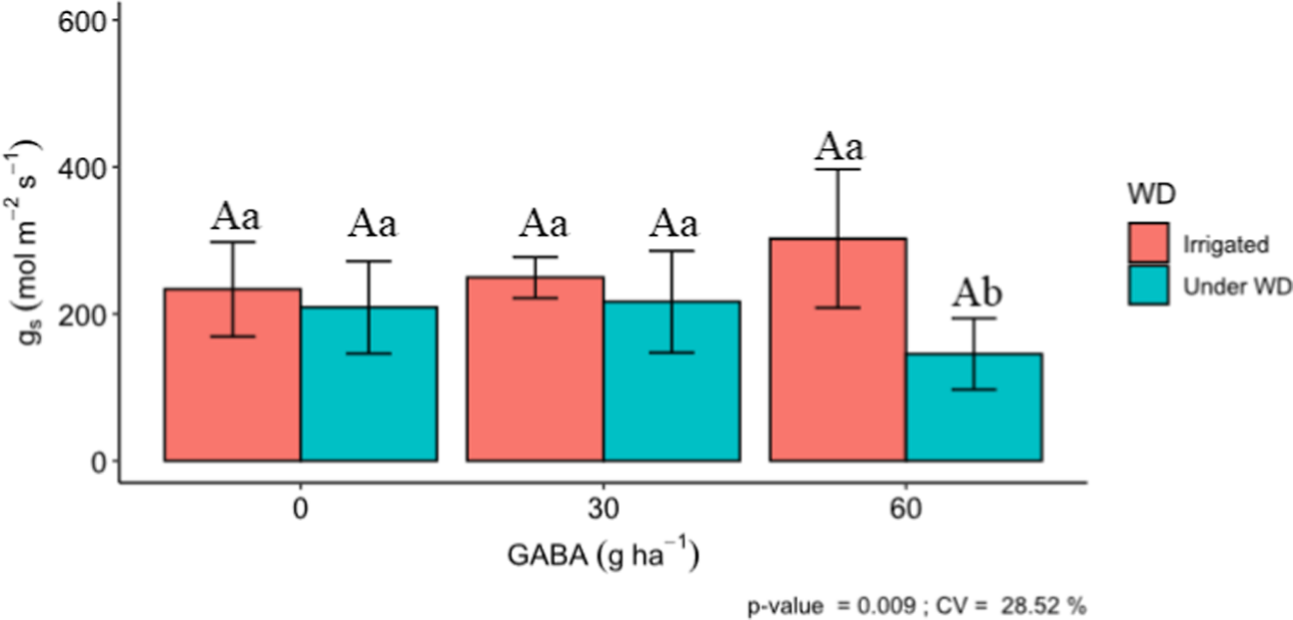
Interaction between foliar application of a biostimulant containing γ-aminobutyric acid (GCB; 0, 30 and 60 g ha^–1^) and water deficit on stomatal conductance (*g*_s_) of maize plants. Data are presented as mean ± standard deviation.

This response suggests that the GCB formulation may contribute to water homeostasis and preservation of leaf physiological function under drought conditions.^13^ Furthermore, effects similar to those observed with other biostimulants include the protection of photosynthetic pigments and structural components, reinforcing the role of these formulations as metabolic stabilizers under stress conditions.^11^ Thus, the responses obtained in the present study indicate that GCB may promote comparable protective mechanisms in maize plants subjected to water deficit.

The similar responses observed between the two GABA doses suggest a physiological saturation point, indicating that 30 g ha^–1^ may be sufficient to achieve the desired effects while being more economically viable.^10,31^ These findings position GCB as a practical and effective tool to enhance the physiological resilience of crops under water stress, with high potential for integration into precision agriculture programs.

PCA scores separated treatments clearly (Figure 5a). WD plants exhibited a tendency to shift to positive PC1, mainly influenced by LT, EL, and RChl, which indicates membrane damage, pigment loss, and stress intensity. These results provide evidence that PC1 captured the drought gradient; irrigated and GCB-treated plants clustered negatively on PC1, associated with higher gs, LA, shoot fresh/dry mass and RWC, reflecting improved water status and growth. Conversely, PC2 represented root development, with negative loadings for RL, RFM, RDM, and SL. GCB-treated plants tended to cluster close to the irrigated controls along PC2 scores, suggesting preservation of root growth and structural balance under WD. These results indicate that WD disrupted cellular homeostasis, while GCB mitigated damage through the combined action of GABA, seaweed-derived hormones, and protein-hydrolysate amino acids, enhancing hydration, stomatal conductance, and biomass allocation.^10,16,18,32^

**Figure 5:**
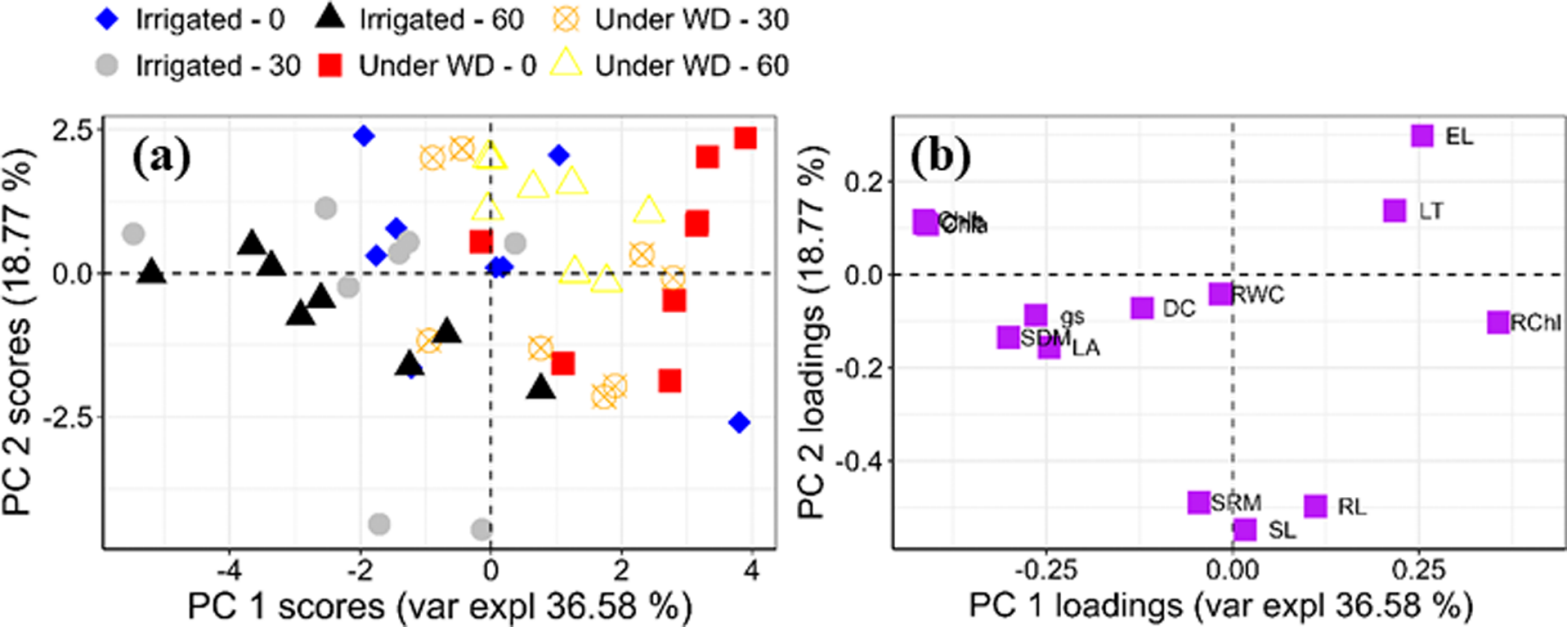
Principal Component Analysis (PCA) of morphophysiological parameters showing the separation of irrigated and water-deficit maize, after between foliar application of a biostimulant containing γ-aminobutyric acid (GCB; 0, 30 and 60 g ha^–1^). Panel (a) shows sample distribution along PC1 and PC2, and panel (b) displays the variable loadings contributing to treatment discrimination.

Different to the above results, the proximity of the treatment clusters with GCB at 30 and 60 g ha^–1^ of GABA to the control group in the PCA scores (Figure 5) indicates that these treatments did not cause marked morphological or physiological deviations, suggesting that the biostimulant helped maintain key physiological processes, such as stomatal conductance and membrane integrity, thereby mitigating oxidative stress.^13,33^ In contrast, the observed tendency of separation among treatments along PC1 (Figure 5a) highlights the physiological and compositional shifts driven by water stress and the modulatory effects of GCB.

Thus, PCA not only highlighted the sensitivity of physiological variables to water deficit but also demonstrated the efficacy of GCB in modulating these responses in a robust manner. These findings reinforce the potential of GCB as an effective strategy for irrigation management, enhancing plant resilience and supporting more sustainable agricultural practices adapted to climate change scenarios.^24,35^

### 3.2 Portable X-Ray Fluorescence Spectral Analysis

Regarding the PCA employed on the spectral pXRF data, two visual approaches were adopted: scores identified only according to the WD treatments (Figure 6a) and samples identified according to the WD treatments but with the addition of 30, and 60 g ha^–1^ of GCB (Figure 6b). In both strategies, the first two components together explained more than 75.0% of the data variance. A qualitative discrimination among treatments was observed along the principal components, indicating distinct multivariate spectral patterns associated with water regime and GCB application (Figure 6a,b), reflecting the physiological and compositional changes induced by water stress and GCB application. From this perspective, while PC1 encompassed the main variation between the treatments, PC2 reflected more subtle variations within the same water conditions. Ashraf et al.,^36^ who reported that the first two PCA dimensions (Dim1 and Dim2) explained 86.1% and 4.0% of the variance in morpho-physiological traits of rice under GCB application and drought stress, respectively.

**Figure 6:**
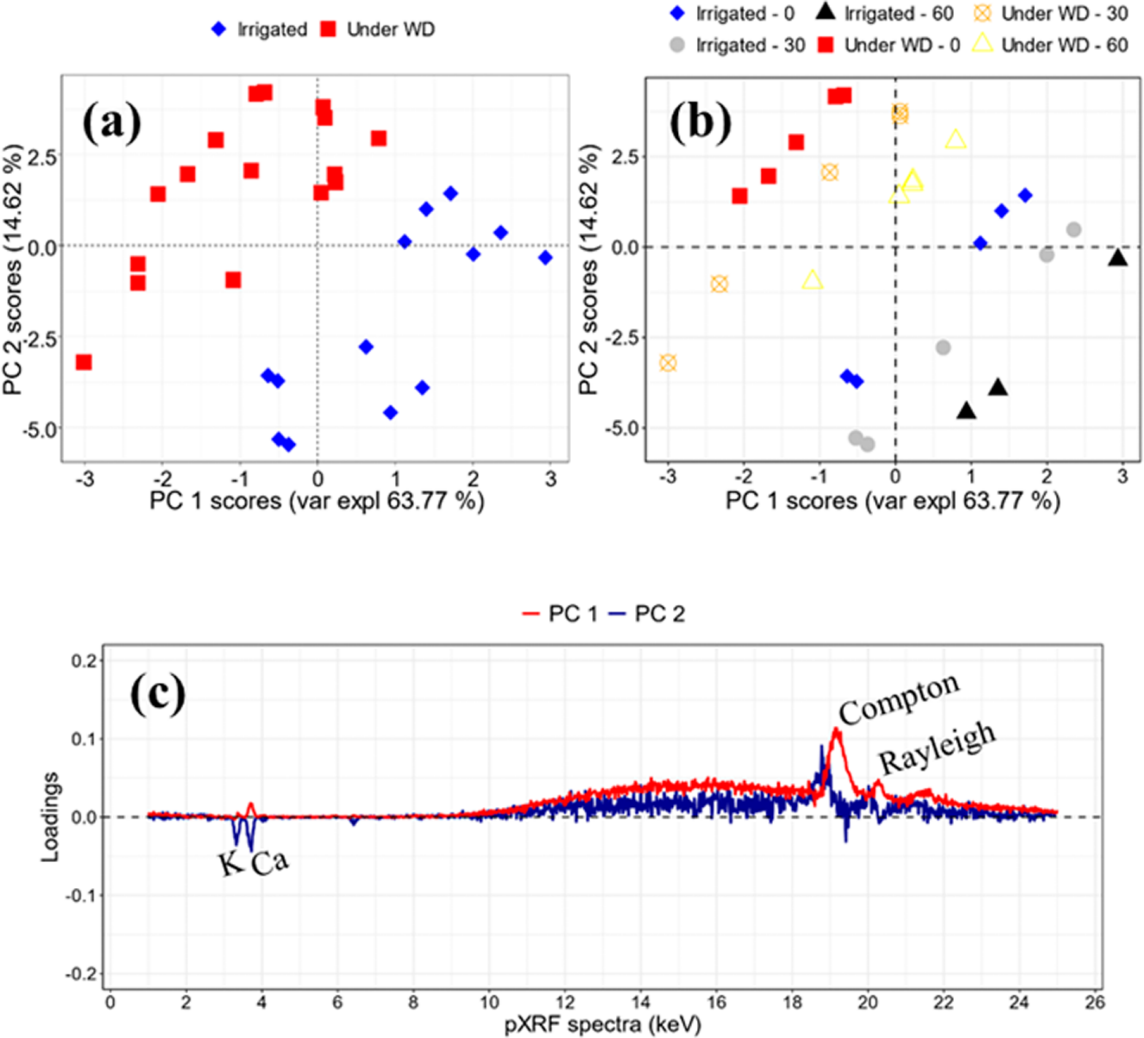
Principal Component Analysis (PCA) of qualitative pXRF spectral responses showing the separation of irrigated and water-deficit maize plants, after between foliar application of a biostimulant containing γ-aminobutyric acid (GCB; 0, 30 and 60 g ha^–1^). Panels (a,b) display sample distribution along PC1 and PC2, and panel (c) shows the spectral regions (K, Ca, Compton and Rayleigh peaks) contributing most to treatment discrimination.

As shown in the score plots of Figure 6a, the observed clustering pattern indicates that the WD treatments tended to yield negative values along the PC1 axis, whereas the irrigated plants tended to cluster in the positive range. Such behavior suggests that PC1 and consequently, the pXRF spectra, effectively captured the difference in leaf water content, being sensitive to cellular dehydration caused by water stress. Meanwhile, adding GCB brought the scores of samples under WD closer to those of the irrigated samples, especially at 60 g ha^–1^ GABA (Figure 6b), suggesting that the biostimulant aided in maintaining leaf hydration through osmotic regulation and stomatal control, as outlined by Xu et al.^15^ and Nasiri et al.^13^

When comparing the results provided by the scores, the analysis of the PC1 loadings underscored the interval between 10 and 24 keV as displaying the highest positive weights along this component, especially the peaks at around 19 to 20 keV, where Rayleigh and Compton scattering predominate (Figure 6c). These characteristic pXRF signals are influenced by Rayleigh and Compton scattering and are sensitive to variations in low atomic number constituents, being indirectly associated with leaf water content and organic composition. Additionally, Costa et al.^22^ observed that this spectral range is an indirect but effective indicator of water content in plant tissues. Accordingly, the tendency for irrigated and GCB-treated WD samples to cluster around positive PC1 values accurately reflects the dehydration caused by water stress and the ability of the biostimulant to modulate this response. Zarbakhsh and Shahsavar^37^ reported enhanced uptake of these minerals in GCB-treated plants, indicating the contribution of GCB to ionic homeostasis under stress. These responses may reflect stress perception and signaling processes, including calcium-related pathways previously described in drought responses, which can contribute to antioxidant regulation and maintenance of cellular turgor under water deficit.^20^

This interpretation aligns with results from Ribeiro et al.,^8^ who demonstrated that X-ray emissions in the 10–24 keV range, especially near 19–20 keV, dominated by Rayleigh and Compton scattering, are highly sensitive to water and organic content in leaf tissues. Their findino confirmed that intact, fresh leaves with higher water content exhibited distinct spectral signatures compared to dehydrated samples. Similarly, Mellouki et al.^23^ highlighted that this spectral region serves as an indirect yet robust indicator of water status in plant tissues, reinforcing the reliability of PC1 in reflecting leaf hydration.

On the other hand, despite explaining a smaller percentage of the total variance, PC2 revealed more subtle differences among water-available treatments, which could be associated with variation in leaf nutritional composition, as the Ca and K emission pXRF lines emerges among the highest PC2 loadings (Figure 6c). These nutrients play a critical role in signaling mechanisms, ion transport, membrane stabilization, and antioxidant defense, all of which are highly regulated under stress conditions.^10,15^

Crespo et al.^38^ provided additional support for multivariate analysis by applying PCA to evaluate antioxidant enzyme activity, osmolyte accumulation, and pigment degradation in *Gmelina arborea* exposed to seasonal water deficits. The authors showed that variables such as proline, peroxidase, and total sugars were inversely correlated with chlorophyll content, and PCA effectively grouped samples by both sampling season and leaf age. This highlights the ability of PCA to integrate diverse biochemical responses into meaningful physiological interpretations, further validating the results observed herein and reinforcing its usefulness in eco-physiological studies under field-relevant conditions.

### 3.3 Colorimetric Analysis

Colorimetric analysis was applied as a rapid and complementary approach to pXRF. It also provided consistent results in distinguishing water deficit conditions and GCB treatments. PCA based on colorimetric scales was performed using the same visualization strategies as for the pXRF data (Figure 7a,b), with the first two principal components explaining over 95.0% of the total variance.

**Figure 7:**
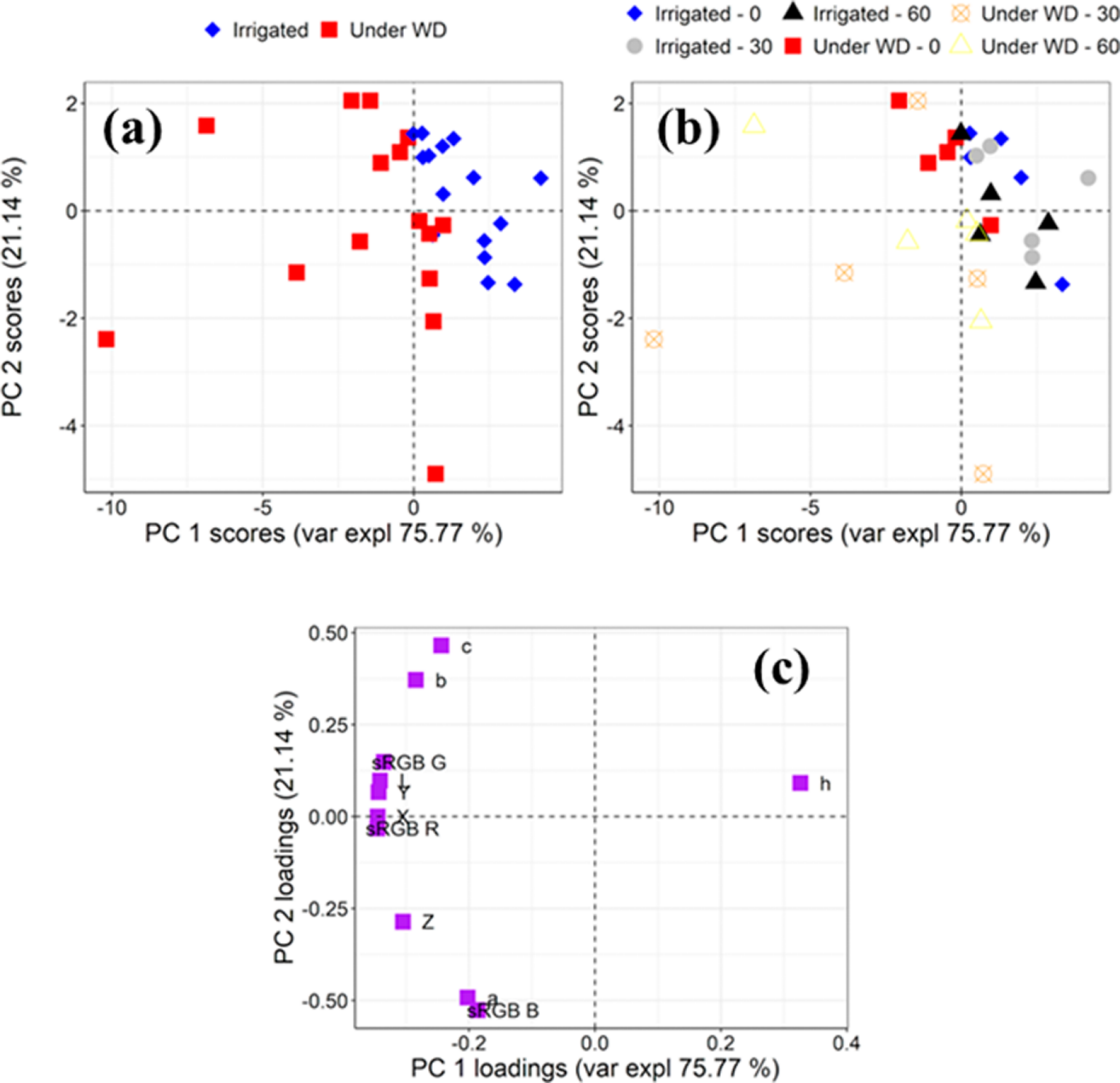
Principal Component Analysis (PCA) of color scales showing the separation of irrigated and water-deficit maize plants, after between foliar application of a biostimulant containing γ-aminobutyric acid (GCB; 0, 30 and 60 g ha^–1^). Panels (a,b) represent sample distribution along PC1 and PC2, while panel (c) shows the variable loadings contributing to treatment discrimination.

Plants under WD and well-irrigated conditions tended to cluster along negative and positive PC1 values, respectively. Among the loadings, hue (h) showed the strongest positive contribution to PC1 (Figure 7c), indicating changes in leaf color tone and saturation. These shifts may reflect alterations in pigment composition and leaf optical properties commonly associated with water stress conditions.^39^

Conversely, other leaf color metrics (such as lightness and chroma) contributed negatively to PC1 and aligned with water-deficit treated samples. These patterns are consistent with the findings of Gutiérrez-Gamboa et al.^40^ who demonstrated in grapevines that RGB-based color indices reliably discriminated differing stress levels under contrasting irrigation regimes.

Despite the strong separation under WD, PCA plots based on GCB treatments (Figure 7b) showed less defined clusters. This may be due to the role of GCB in modulating pigment retention and antioxidant activity, which can blur stress-induced color distinctions.^31,41^ The variability suggests that while GCB enhances physiological resilience, its protective effects may reduce colorimetric sensitivity as a stress marker.

### 3.4 Visible Color Spectra (400–700 nm)

The PCA involving the visible-range spectra (400–700 nm), aligned with previous visual approaches, is presented in Figure 8. The first two components explained more than 95.0% of the total variance, capturing the most significant differences among the sample groups.

**Figure 8:**
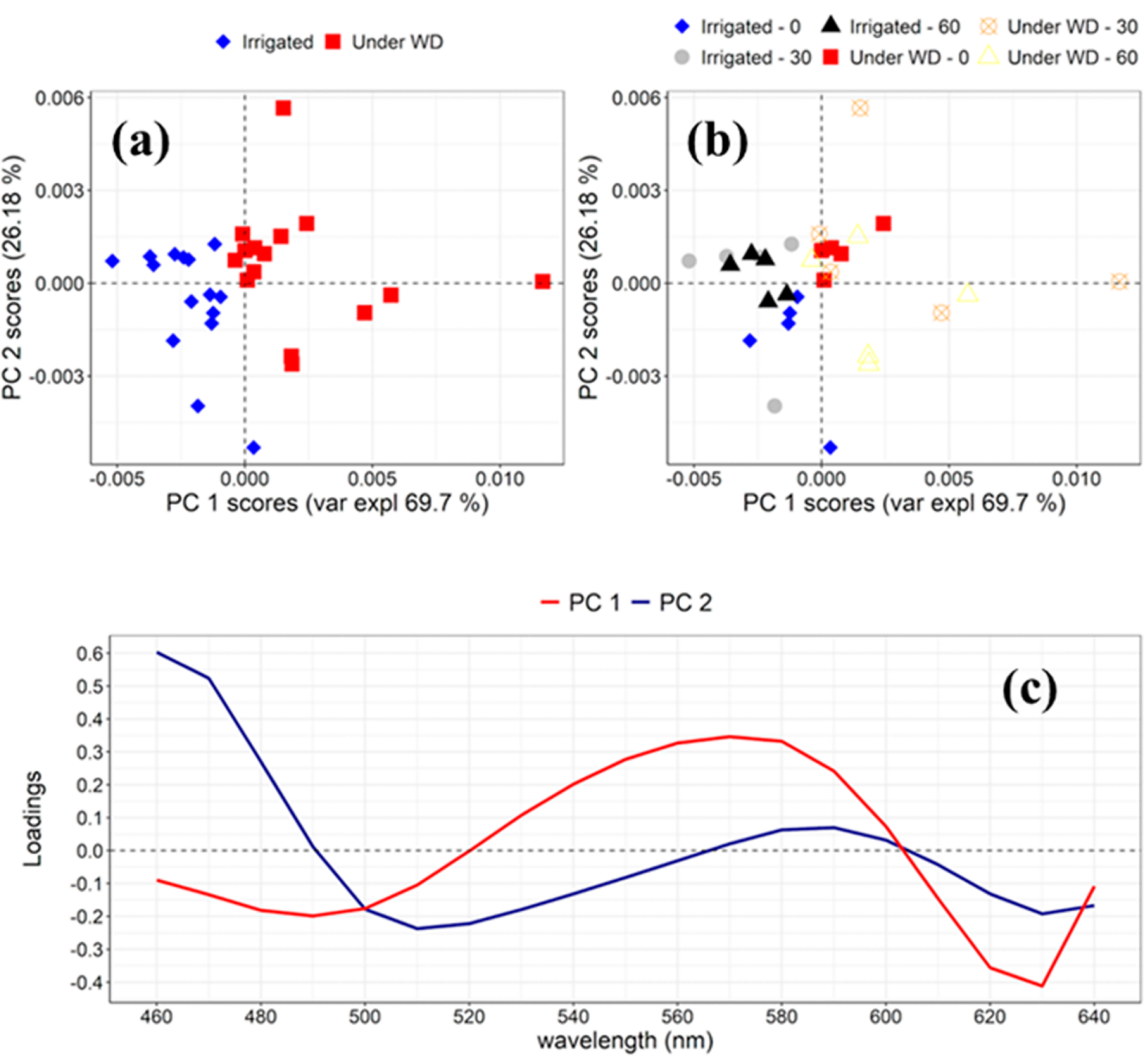
Principal Component Analysis (PCA) of the visible spectrum for showing the separation of irrigated and water-deficit maize plants, after between foliar application of a biostimulant containing γ-aminobutyric acid (GCB; 0, 30 and 60 g ha^–1^). Panels (a,b) represent sample distribution along PC1 and PC2, while panel (c) shows the variable loadings contributing to treatment discrimination.

The score plots (Figure 8a) also showed a clear clustering tendency, separating irrigated and WD samples primarily along PC1. Irrigated plants exhibited negative PC1 scores, while WD samples were mostly positioned on the positive side. According to the loadings (Figure 8c), the 520–600 nm band, associated with green, yellow, and a small portion of orange, had the highest positive influence on PC1, reflecting increased absorbance in this spectral region by plants under WD. Although this region is typically characterized by low chlorophyll absorption and higher reflectance in healthy plants, the increase in absorbance may indicate structural changes or pigment degradation, such as reductions in chlorophyll or alterations in leaf mesophyll, consistent with previous findings under drought stress.^39,42^

Additionally, the 460–520 nm and 600–640 nm bands, associated with blue, and a small interval of green, orange, and red light, exhibited negative loadings along PC1 and were more closely associated with irrigated samples. These spectral regions are influenced by carotenoids, chlorophyll *b*, and other accessory pigments. The stronger reflectance or lower absorbance observed under irrigation conditions may reflect the preservation of pigment integrity and photoprotective function. In contrast, their reduced expression among WD samples is consistent with pigment degradation under drought stress. According to Carter and Spiering,^43^ wavelengths in the 550–625 nm range are particularly sensitive to changes in leaf chlorophyll content. Therefore, reductions in reflectance within this region, often observed under water deficit conditions, may be associated with decreases in chlorophyll and accessory pigments, although further physiological confirmation is required.

Interestingly, some of the WD samples with GCB applied, particularly those in the 60 g ha^–1^ group, shifted closer to the irrigated cluster in Figure 8b, approaching PC1 zero-value scores. This trend suggests that GCB contributes to photoprotection and reduces pigment damage under drought conditions, likely through enhanced antioxidant activity, osmotic regulation, and stabilization of pigment protein complexes.^33^ These findings underscore the role of GCB in improving drought resilience and support the use of visible-range spectral analysis as a noninvasive tool to monitor physiological integrity under environmental stress.^31^

### 3.5 Final Remarks

The current study demonstrates the effectiveness of integrating physiological, structural, and spectral data to evaluate the impacts of WD and the mitigating potential of γ-aminobutyric acid- containing biostimulant (GCB) in maize plants. Across multiple analytical approaches, including ANOVA, PCA, and spectral analyses in the visible spectroscopy and X-ray fluorescence domains, WD was consistently shown to reduce key growth parameters, chlorophyll content, and stomatal conductance, while increasing leaf temperature and membrane damage, hallmarks of oxidative and osmotic stress (30–10).

GCB application, especially at 30 and 60 g ha^–1^, proved effective in counteracting these stress effects, promoting increased biomass allocation, maintained pigment integrity, and stabilized stomatal conductance, and reinforcing its role as a multifunctional biostimulant.^10,34^ These physiological benefits were mirrored in the spectral domains: pXRF revealed GCB-induced clustering of WD samples closer to irrigated plants, due to improved hydration status,^8^ while visible-range spectra indicated preserved pigment absorption patterns in the green–yellow region (490–600 nm) and attenuated degradation in the chlorophyll-associated red band (670–690 nm).^39,42^

The multivariate analysis also revealed that PCA is a powerful tool for integrating high-dimensional data to track stress-induced alterations and GCB-mediated adaptations.^37^ Spectral PCA, in particular, captured subtle shifts in water status, pigment degradation, and structural responses associated with different stress levels. These findings support the use of hyperspectral and sensor-based phenotyping techniques in precision agriculture.^24^

By demonstrating the capacity of GCB to preserve physiological function and promote growth under drought, the current work contributes to the growing evidence base positioning GCB as a practical, scalable strategy in climate-resilient crop management. Its application may serve not only to mitigate yield losses under water-limited conditions but also to enhance overall plant robustness and performance across variable environmental contexts.^11,13,33^

The water deficit significantly compromised the growth, water status, cell membrane integrity, and photosynthetic pigment stability of corn plants. Foliar application of GCB mitigated these adverse effects by promoting greater water retention, maintaining stomatal conductance, preserving pigment integrity, and strengthening the plant’s structure, even under severe stress conditions.

Portable X-ray fluorescence and visible spectroscopy analyses combined with principal component analysis demonstrated sensitivity in detecting early physiological and biochemical changes.

Specifically, the PCA-pXRF approach discerned both water deficit and alterations induced by variable GCB application. Notably, these responses were predominantly attributed to Rayleighvest and Compton scattering, as well as the characteristic pXRF emissions of K and Ca. Conversely, the PCA approaches via visible spectrum and color scales were consistent in discriminating samples according to water deficit (attributed mainly to the 500–600 nm band and the h parameter, respectively); however, they were less sensitive to differentiating GCB applications.

Overall, the integration of both spectral analysis with PCA proved to be a robust, nondestructive, and fast approach for physiological monitoring with great potential for field use in precision management strategies with real-time responses. Meanwhile, integrating biostimulants, such as GCB, with portable spectral devices is a promising strategy for enhancing crop resilience to water stress and enabling more sustainable, efficient precision management in the face of agricultural challenges posed by climate change.

## Supplementary Material



## Notes

Additional images of the nondestructive spectral acquisition procedure using portable X-ray fluorescence (pXRF) in maize leaves and amino acid composition of the collagen hydrolysate (PDF)

## Abbreviations

WD: water deficit
GCB: GABA-containing biostimulant
GABA: γ-aminobutyric acid
pXRF: portable X-ray fluorescence
PCA: principal component analysis
RWC: relative water content
TM: turgid mass
DM: dry mass
FM: fresh mass
EL: electrolyte leakage
Chl a: chlorophyll a
Chl b: chlorophyll *b*
Chl t: total chlorophyll
RChl ab: chlorophyll a/b ratio
LT: leaf temperature
*g*_s_: stomatal conductance
SD: stem diameter
SL: shoot length
RL: root length
LA: leaf area
SFM: shoot fresh mass
RFM: root fresh mass
SDM: shoot dry mass
RDM: root dry mass
PC1, PC2: principal components 1 and 2
RGB: red, green, blue
XYZ: tristimulus color space
Lab: color space L*, a*, b*
LCh: color space lightness, chroma, hue
Tracer 5i: pXRF spectrometer (Bruker)
NIX Pro 2: digital colorimeter
CFL1030: clorofilômetro falker
UTi-260B: Uni-T thermal camera
SC1: decagon porometer
LI-3000C: LI-COR electronic leaf area meter
ANOVA: analysis of variance
F-test: teste F
C.V.: coefficient of variation
