# Supplementary material for: Effects of Water Deficit and GABA-Containing Biostimulant on Maize Plants: Nondestructive Monitoring by X‑Ray Fluorescence and Visible Spectroscopy
Source: ACS Omega. 2026 Jun 2;11(23):33775–86. doi: 10.1021/acsomega.6c00213 (PMC13280822; doi:10.1021/acsomega.6c00213)

Supporting Information

Effects of water deficit and GABA-containing biostimulant on maize plants: Non-destructive monitoring by X-ray fluorescence and visible spectroscopy.

Authors

João Pedro Chacon Pereira[1]

Fábio Luiz Melquiades[2]

José Vinícius Ribeiro[2]

Gabriela Machineski[1]

Inês Cristina de Batista Fonseca[1]

Halley Caixeta Oliveira[3]*

Affiliations

[1] Department of Agronomy, Postgraduate Program in Agronomy, State University of Londrina (UEL), 86057-970, Londrina, PR, Brazil

[2] Department of Physics, State University of Londrina (UEL), 86057-970, Londrina, PR, Brazil

[3] Department of Animal and Plant Biology, State University of Londrina (UEL), 86057-970, Londrina, PR, Brazil

Corresponding author: Halley Caixeta Oliveira (halley@uel.br)

Table S1. Amino acid composition and free amino acid profile of the collagen hydrolysate used in the experiment. Data adapted from Eurofins (2023).

**Amino Acid Profile**			**Free Amino Acid Profile**		
**Parameter**	**Results**	**Unit**	**Parameter**	**Results**	**Unit**
Aspartic Acid	3,07	%	Aspartic Acid	0,05	%
Glutamic Acid	5,62	%	Glutamic Acid	0,09	%
Alanine	5,73	%	Alanine	0,32	%
Arginine	2,02	%	Arginine	<0,01	%
Phenylalanine	1,12	%	Phenylalanine	0,03	%
Glycine	13,49	%	Glycine	1	%
Histidine	0,41	%	Histidine	<0,01	%
Isoleucine	0,75	%	Isoleucine	0,88	%
Leucine	1,82	%	Leucine	<0,01	%
Lysine	1,85	%	Lysine	0,03	%
Proline	8,26	%	Proline	0,22	%
Serine	0,36	%	Serine	0,24	%
Tyrosine	<0,04	%	Tyrosine	<0,01	%
Threonine	0,23	%	Threonine	0,01	%
Valine	1,49	%	Valine	<0,01	%
Cystine + Cysteine	0,03	%	Cystine	0,02	%
Methionine	0,54	%	Methionine	0,07	%
Collagen	360	mg/g	Collagen	360	mg/g

Figure S1. Experimental setup for non-destructive spectral acquisition in maize leaves using portable X-ray fluorescence (pXRF): (a) direct positioning of the maize leaf for spectral acquisition, (b) use of support material during spectral measurements, and (c) overall configuration of the pXRF measurement system under greenhouse conditions.

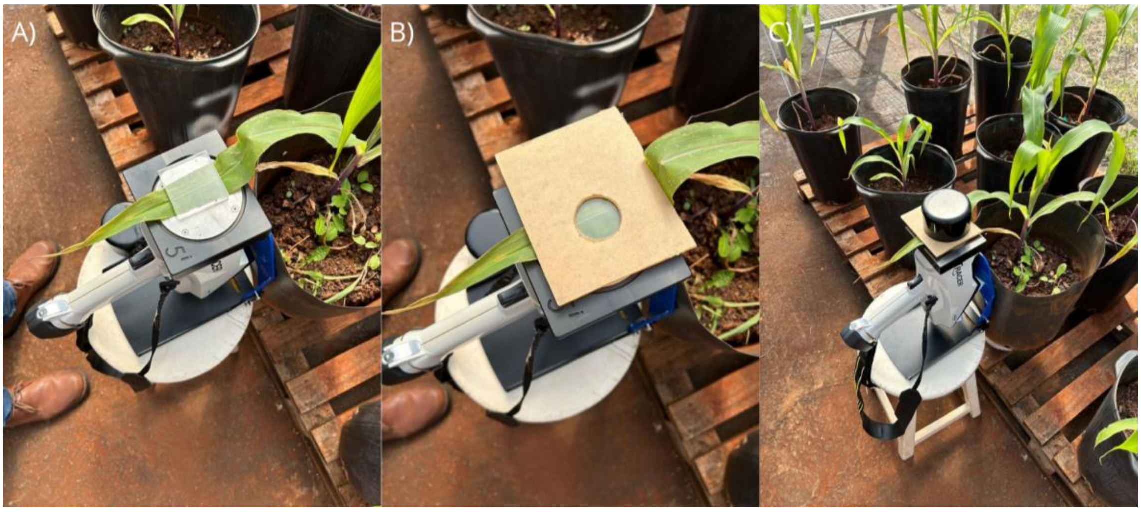

Supplement: Supplementary file 1 [file ao6c00213_si_001.pdf]
